# Supplementary material for: Loss of Propionyl-CoA Carboxylase Reprograms Hepatic Metabolism by Suppressing Mitochondrial Pyruvate Carboxylation and Fatty Acid Oxidation
Source: bioRxiv. 2026 Apr 15:2026.04.13.718201. Preprint. [Version 1] doi: 10.64898/2026.04.13.718201 (PMC13104956; doi:10.64898/2026.04.13.718201)
Supplement: Supplement 1 [file NIHPP2026.04.13.718201v1-supplement-1.pdf]

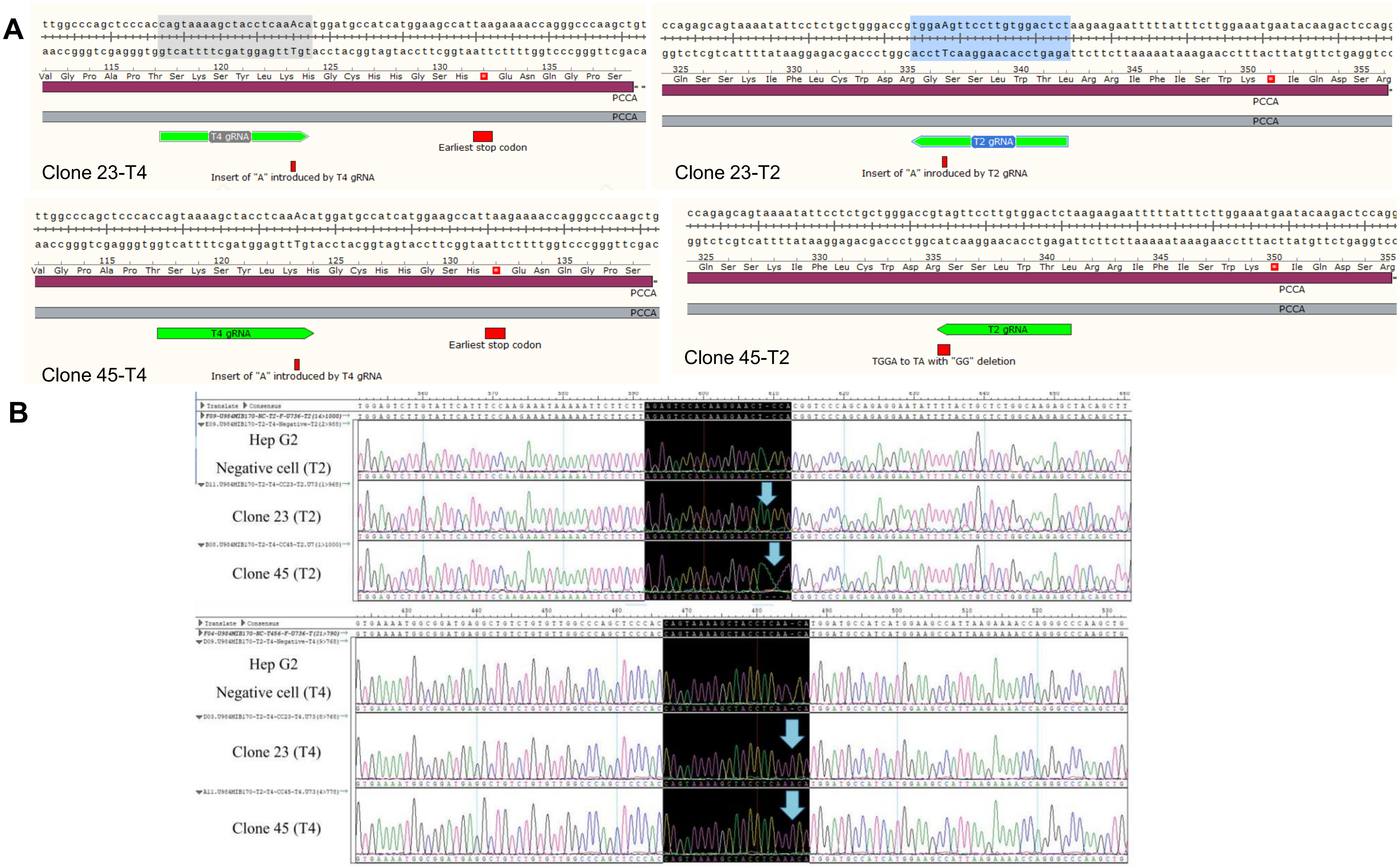

Supplementary Figure 1. Biallelic knockout of the *PCCA* gene in HepG2 cells. (A) Schematic representation of the guide RNAs and the resulting mutations in two isogenic knockout clones. (B) Sanger sequencing traces of the two isogenic knockout clones compared with parental HepG2 cells.

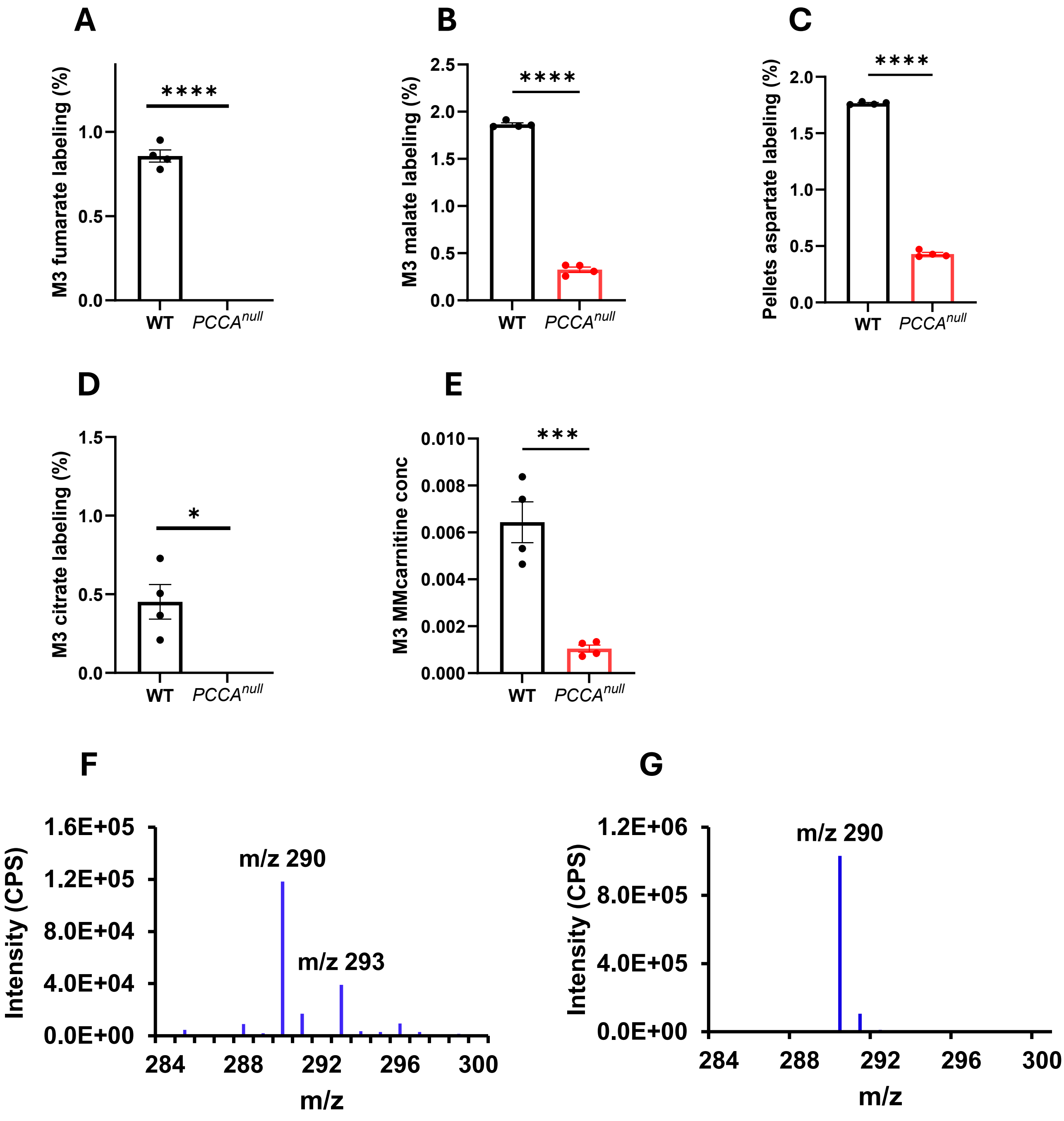

Supplementary Figure 2. Reduced propionyl-CoA metabolism in *PCCA*^{null}-HepG2 cells. (A–D) M3 isotopomer labeling of fumarate, malate, aspartate, and citrate in wild-type (WT) and *PCCA*^{null}-HepG2 cells following incubation with 1 mM [$^{13}C_3$]propionate for 4 hours. (E) Abundance of M3 methylmalonylcarnitine (MM-carnitine) in WT and *PCCA*^{null}-HepG2 cells after 4-hour incubation with 1 mM [$^{13}C_3$]propionate. (F–G) Mass spectra of methylmalonylcarnitine in HepG2 cells cultured with or without [$^{13}C_3$]propionate. Data are presented as mean ± SEM (n = 4). *, ***, and **** indicate $p < 0.05$, $p < 0.005$, and $p < 0.0001$, respectively.

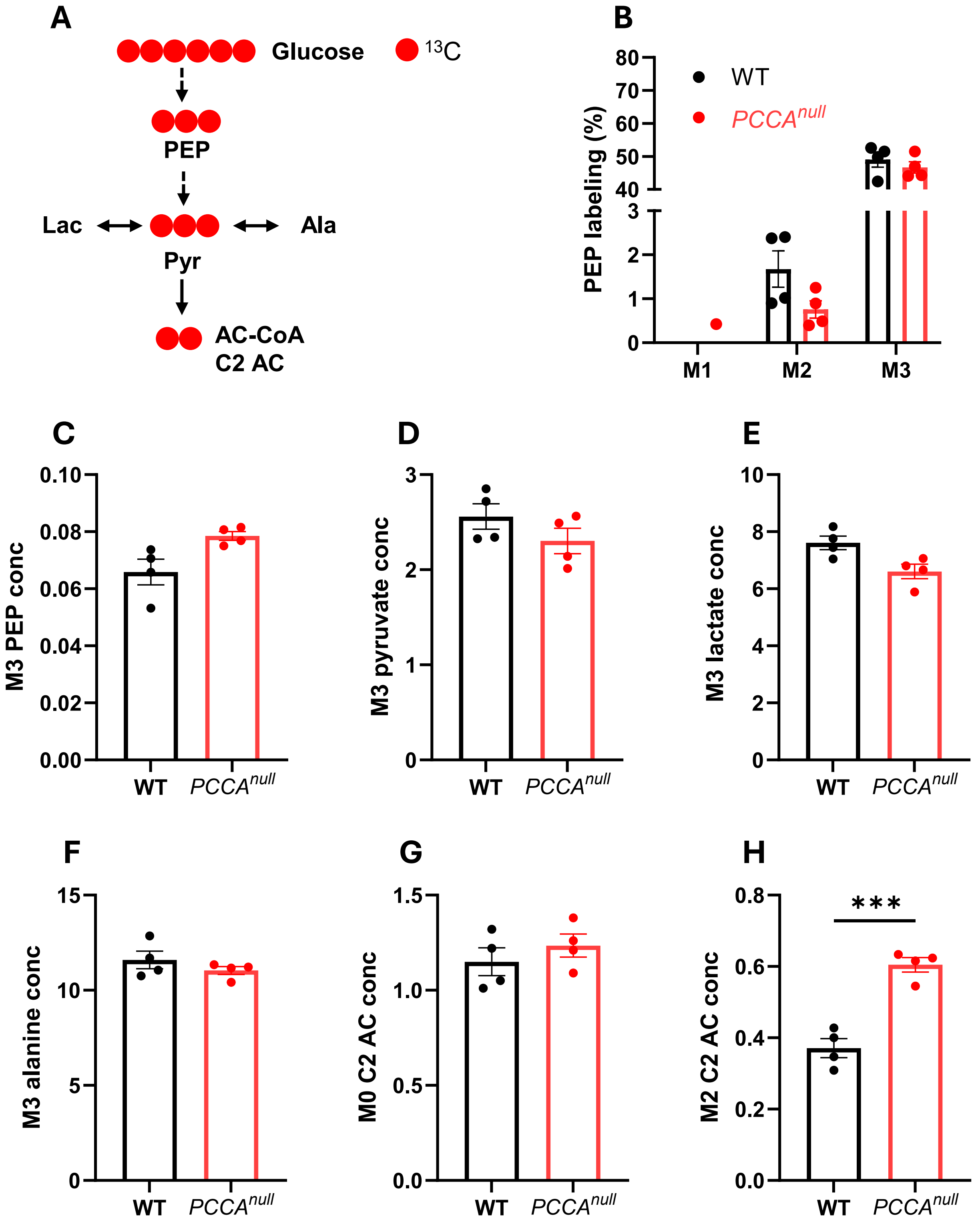

Supplementary Figure 3. *PCCA* knockout enhances glucose metabolism through PDH. (A) Schematic of glycolysis and the generation of acetyl-CoA/acetylcarnitine from [$^{13}C_6$]glucose. (B) Isotopomer distribution of phosphoenolpyruvate (PEP). (C–H) Levels of M3 PEP, M3 pyruvate, M3 lactate, M3 alanine, M0 acetylcarnitine, and M2 acetylcarnitine in wild-type (WT) and *PCCA*^{null}-HepG2 cells. Data are presented as mean ± SEM (n = 4). **** indicates p < 0.0001.

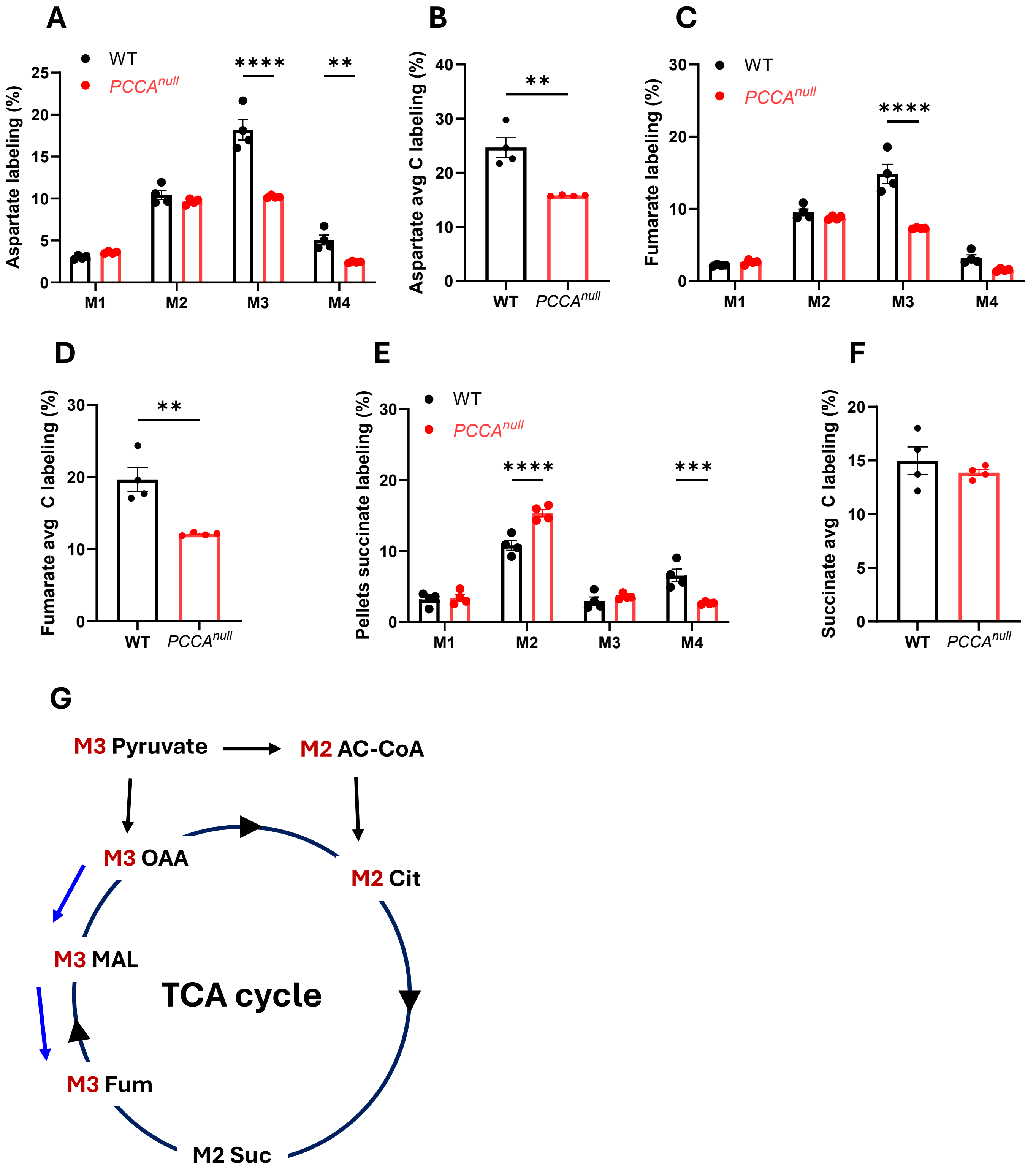

Supplementary Figure 4. ***PCCA*** **knockout reduces pyruvate anaplerosis in HepG2 cells**. (A–F) Mass isotopomer distributions and average carbon (avg C) labeling of aspartate, fumarate, and succinate in wild-type (WT) and *PCCA*^{null}-HepG2 cells following incubation with 11 mM [$^{13}C_6$]glucose for 4 hours. (G) Schematic illustrating the predominant isotopomers of TCA cycle intermediates derived from [$^{13}C_6$]glucose via pyruvate carboxylation and pyruvate dehydrogenase flux. Data are presented as mean ± SEM (n = 4). ** and **** indicate $p < 0.01$ and $p < 0.0001$, respectively.

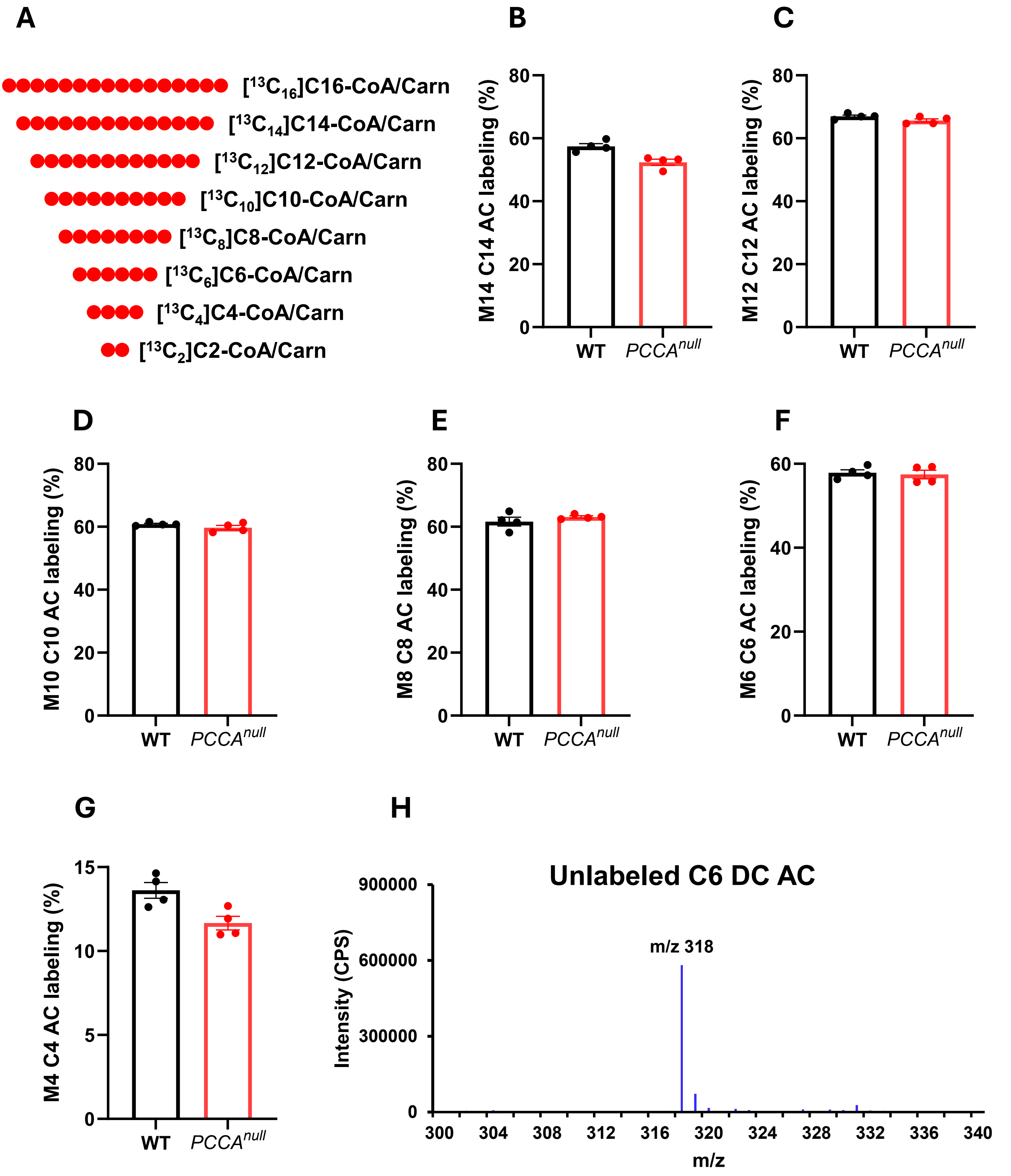

Supplementary Figure 5. Unchanged acylcarnitine labeling derived from [$^{13}C_{16}$]palmitate in *PCCA^{null}*-HepG2 cells. (A) Schematic of acylcarnitine intermediates generated along the fatty acid β-oxidation pathway. (B–G) Labeling of M14 C14 acylcarnitine (C14 AC), M12 C12 AC, M10 C10 AC, M8 C8 AC, M6 C6 AC, and M4 C4 AC in wild-type (WT) and *PCCA^{null}*-HepG2 cells following incubation with 0.4 mM [$^{13}C_{16}$]palmitate for 4 hours. (H) Mass spectrum of unlabeled C6 dicarboxylylcarnitine (C6 DC AC). Data are presented as mean ± SEM (n = 4).

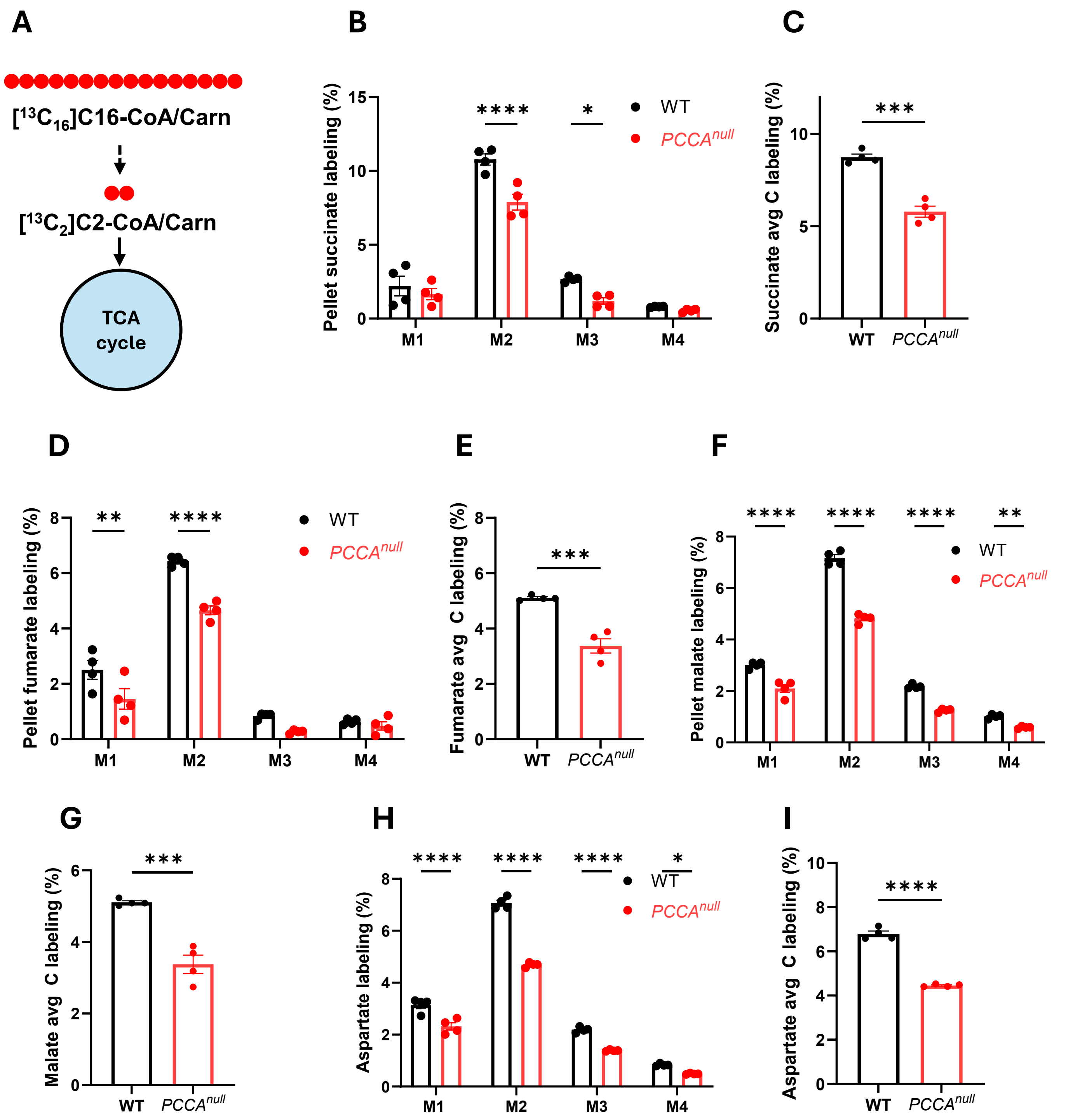

Supplementary Figure 6. Reduced mitochondrial fatty acid oxidation in *PCCA^{null}*- HepG2 cells. (A) Schematic of [$^{13}C_{16}$]palmitate metabolism entering the TCA cycle. (B–I) Stable isotopomer distributions and average carbon (Avg C) labeling of succinate, fumarate, malate, and aspartate in wild-type (WT) and *PCCA^{null}*-HepG2 cells following incubation with 0.4 mM [$^{13}C_{16}$]palmitate for 4 hours. Data are presented as mean ± SEM (n = 4). *** and **** indicate $p < 0.005$ and $p < 0.0001$, respectively.

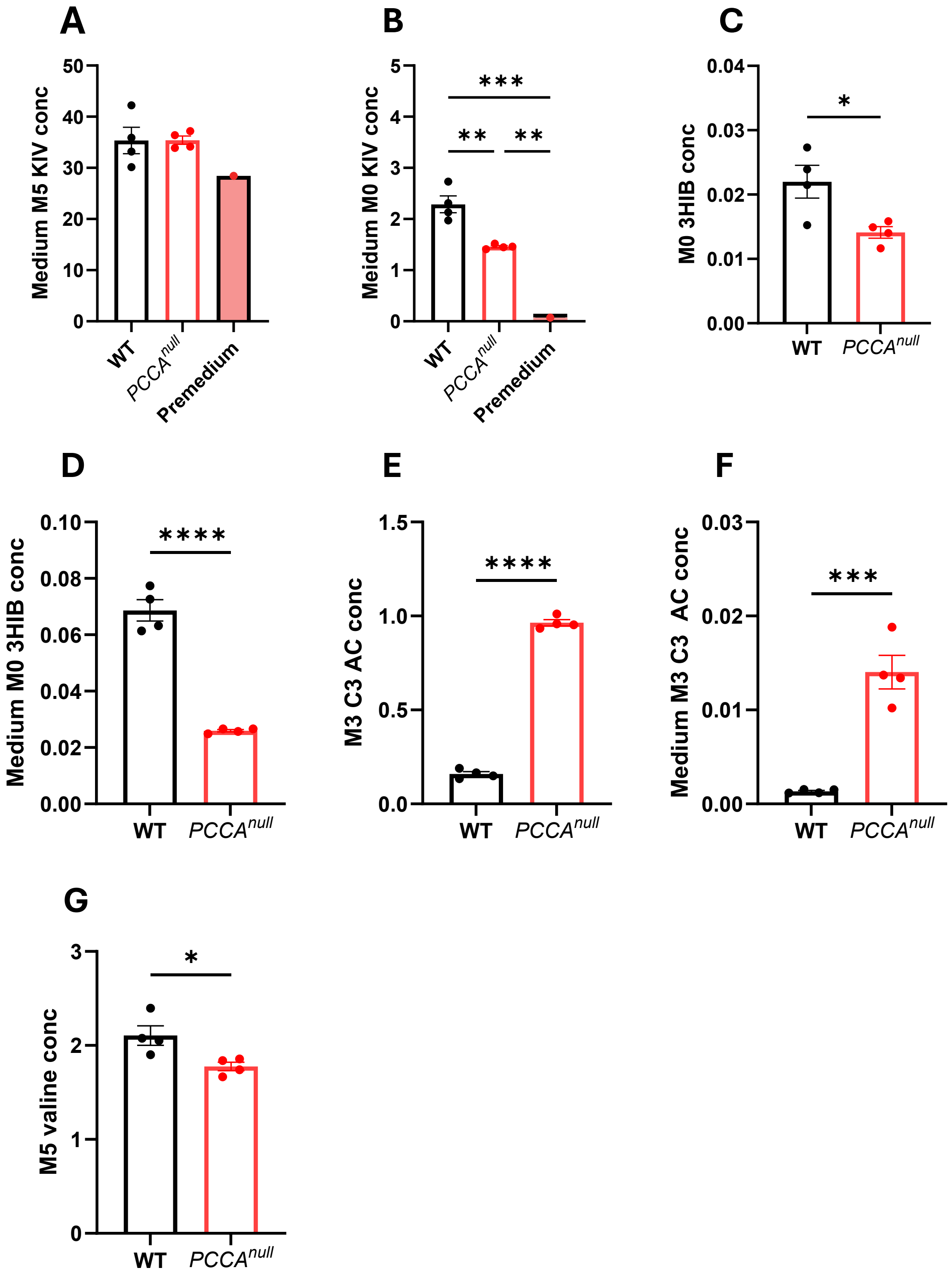

Supplementary Figure 7. PCCA deficiency reduces branched-chain keto acid (BCKA) catabolism. (A–B) M5 and M0 KIV levels in pre-incubation and conditioned media from WT and *PCCA*^{null}-HepG2 cells. (C–F) Unlabeled 3-hydroxyisobutyrate (3-HIB) and M3 propionylcarnitine (C3-carnitine) levels in cell pellets and culture media. (G) M5 valine levels in WT and *PCCA*^{null}-HepG2 cells. Data are shown as mean ± SEM (n = 5). *P < 0.05, **P < 0.01, ***P < 0.005, ****P < 0.0001.

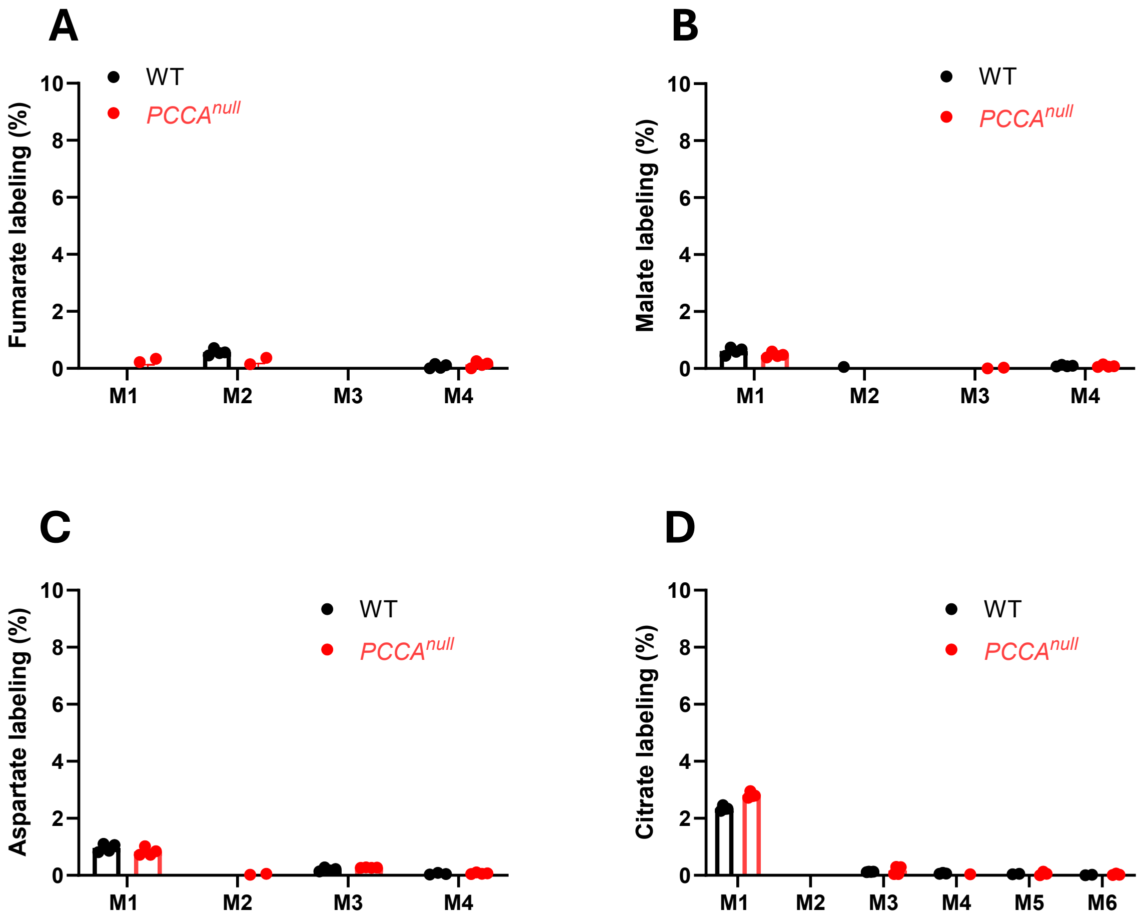

Supplementary Figure 8. Minimal metabolism of threonine in HepG2 cells. (A–D) Stable isotopologue labeling of fumarate, malate, aspartate, and citrate in WT and *PCCA*^{null}-HepG2 cells following treatment with 0.5 mM [$^{13}C_4$]threonine for 4 h. Data are shown as mean ± SEM (n = 4).
